# Prevalence of dry eye symptoms and associated risk factors among students at Al-Mustaqbal University, Iraq

**DOI:** 10.1371/journal.pone.0337335

**Published:** 2025-12-19

**Authors:** Hassan A. Aljaberi, Saeed Rahmani, Zainab A. Elzahra, Samir H. Mohammed

**Affiliations:** 1 Optical Techniques Department, College of Health and Medical Techniques, Al-Mustaqbal University, Babylon, 51001, Iraq; 2 Department of Optometry, Faculty of Rehabilitation, Shahid Beheshti University of Medical Sciences, Tehran, Iran; 3 Department of Vision Screening Techniques, College of Health and Medical Techniques, Al-Furat Al-Awsat Technical University, An-Najaf, Iraq; Canadian University Dubai, UNITED ARAB EMIRATES

## Abstract

This study aimed to estimate the prevalence, severity, and risk factors of dry eye symptoms (DES) among Iraqi university students and to examine associations with digital device use in an arid, dust-prone setting. A cross-sectional survey was conducted at Al-Mustaqbal University (Iraq) between February and May 2025 using paper-based questionnaires that captured demographics, device-use patterns, symptom burden, and self-management behaviors. Symptom severity was assessed with the Ocular Surface Disease Index (OSDI), and DES prevalence was estimated using the Women’s Health Study (WHS) questionnaire. Binary logistic regression was applied to calculate adjusted odds ratios (aORs) with 95% confidence intervals (CIs). Among 1,487 invited students, 1,154 completed the survey (response rate 77.6%); participants were aged 19–24 years, and 63.6% were female. The WHS-defined prevalence of symptomatic DES was 62.0%. Blurred vision, burning, and ocular redness were the most frequent symptoms. A dose–response relationship was observed between daily screen time and OSDI severity, with the highest burden among students reporting more than 7 hours per day (trend p < 0.001). In adjusted models, female participants had higher odds (aOR 2.34; 95% CI 1.56–3.56; p < 0.001), screen time >7 h/day (aOR 2.25; 95% CI 1.55–3.31; p < 0.001), fourth academic year (aOR 1.75; 95% CI 1.18–2.60; p = 0.01), and use of glasses/contact lenses (aOR 1.45; 95% CI 1.10–1.90; p = 0.01) were significant predictors. Laptops were most often associated with ocular redness, smartphones with burning and pain, and tablets with blurred vision. In conclusion, DES symptoms were highly prevalent and strongly associated with prolonged daily screen exposure. Female sex, advanced academic standing, and corrective lens use further increased risk. Findings support campus-level prevention focused on digital hygiene, ergonomic optimization, and periodic ocular screening, considering environmental stressors typical of arid, dust-prone settings.

## Introduction

Dry Eye Syndrome (DES) is a multifactorial disorder of the tear film and ocular surface that causes ocular discomfort, visual disturbances, and in severe cases, damage to the ocular surface [1–5]. The cornea is covered by a thin, multilayered tear film composed of lipid, aqueous, and mucin components that collectively lubricate, nourish, and protect the ocular surface [2,6]. Disruption of this delicate equilibrium leads to increased tear film osmolarity, inflammation, and cellular stress, which impair the lacrimal and meibomian gland functions responsible for aqueous and lipid secretion [3,4].

The clinical manifestation of DES varies widely, but symptoms are the primary determinants of disease burden. Patients frequently report burning, grittiness, ocular redness, blurred vision, photophobia, tearing, and dryness [7,8]. These symptoms affect daily tasks such as reading, computer work, and driving, and they contribute to decreased quality of life and academic productivity. Persistent symptoms can also lead to psychological fatigue and increased healthcare utilization [9,10]. Among younger populations, especially university students, these symptoms may compromise academic performance and long-term visual health.

A wide range of intrinsic and extrinsic factors contribute to DES. Intrinsic factors include advancing age, female sex, hormonal imbalance, and systemic diseases such as diabetes and thyroid dysfunction [11,12]. Extrinsic risk factors include the use of topical or systemic medications, nutritional deficiencies (e.g., vitamin A), smoking, and extensive visual display terminal (VDT) use [9,13,14]. Environmental exposures also play a crucial role: low ambient humidity, high air pollution, wind, dust, and ultraviolet radiation can destabilize the tear film and accelerate evaporation [12,15]. The extensive use of digital devices smartphones, tablets, and laptops, has emerged as a leading contributor to ocular discomfort worldwide. Prolonged screen exposure reduces blink rate, increases incomplete blinking, and enhances tear evaporation, all of which exacerbate ocular surface stress [9,14]. During and after the COVID-19 pandemic, this risk has intensified due to the rise in online learning and prolonged indoor screen exposure [16–19]. Additionally, blue light emissions from digital displays have been implicated in further destabilizing the tear film and causing oxidative stress on the ocular surface [20,21].

Environmental and climatic factors substantially influence DES prevalence. Arid climates, high temperature fluctuations, and recurrent dust storms are common throughout the Middle East and are known to increase tear evaporation and ocular surface irritation [15,22]. In Iraq, these environmental conditions are particularly pronounced, leading to increased exposure to airborne particulate matter and extended periods of low relative humidity. Such factors may compound the effects of behavioral risks such as screen overuse, especially among young adults. Comparable studies from regional settings, including Saudi Arabia, Jordan, and Iran, have reported similarly elevated DES prevalence linked to dust exposure, pollution, and indoor air conditioning [23–26]. These findings highlight the relevance of environmental stressors alongside behavioral patterns in shaping DES risk profiles across the Middle East.

Despite the growing body of international literature, there remains a paucity of data on DES in Iraq. Considering the unique environmental, social, and academic contexts of Iraqi students, this study aims to estimate the prevalence, severity, and risk factors of DES among university students at Al-Mustaqbal University. The research further explores associations between digital device use and ocular symptoms in a region characterized by arid and dust-prone conditions, with the goal of informing public health strategies that promote ocular health and digital hygiene among young adults.

## Materials and methods

### Study design

A cross-sectional observational study was conducted to evaluate the impact of digital device usage on dry eye symptoms among university students. The study was carried out at Al-Mustaqbal University, Iraq, from February 1, 2025, to May 31, 2025. Data collection was performed using paper-based questionnaires to ensure accessibility and minimize bias associated with online surveys.

### Study population

The study targeted 1,487 students from multiple academic departments, with no restrictions on age or educational level. A total of 1,154 students completed the questionnaires, achieving a 77.6% response rate, which provided a robust sample size for statistical analyses.

Previous institutional research at Al-Mustaqbal University has examined ocular parameters among the same population, further supporting the relevance of this group for visual health research [27].

### Sampling procedure

The sample was selected using a convenience sampling technique, as participation was limited to students who were available and willing to take part during data collection.

The required sample size was estimated using the Cochran formula and recent methodological guidance for cross-sectional survey research [28,29].


$$n=\frac{Z^{\text{2}}P(\text{1}-P)}{d^{\text{2}}}$$ (1)

where n is the minimum required sample, Z = 1.96 for a 95% confidence interval, P = 0.5 (expected prevalence), and d = 0.05 (margin of error). This calculation yielded a minimum required sample of approximately 384 students; thus, the achieved sample (n = 1,154) exceeded the requirement, ensuring adequate statistical power.

### Questionnaire tools

Two validated assessment instruments were used to evaluate dry eye symptoms. The Ocular Surface Disease Index (OSDI) was used to quantify symptom frequency and severity, and the Women’s Health Study (WHS) questionnaire was used to estimate the prevalence of DES. The validated Arabic version of the OSDI was applied as previously reported in the literature [30,31]. These standardized tools ensured reliable assessment of symptom burden and ocular health status among participants.

### Statistical analysis

All analyses were performed using SPSS software, version 28 (IBM Corp., Chicago, IL, USA). Categorical variables were summarized as frequencies and percentages, while binary logistic regression identified risk factors associated with dry eye symptoms. Odds ratios (ORs) with 95% confidence intervals (CIs) were calculated, and a p-value < 0.05 was considered statistically significant.

### Ethics statement

The study protocol was reviewed and approved by the Research Ethics Committee of Al-Mustaqbal University, Iraq (Approval No. 2561/2025, dated 02 January 2025). All participants were aged ≥18 years and provided voluntary participation. The ethics statement was updated to indicate that students received an informed consent form explaining the study objectives, confidentiality, and voluntariness. Completing and returning the questionnaire constituted implied consent.

## Results

### Demographic characteristics

A total of 1,154 students, ages 19–24, participated in this study. Female participants represented 63.6% (n = 734), while males accounted for 36.4% (n = 420). The majority of respondents were enrolled in the Optical Techniques Department (55.6%), followed by Medical Laboratory Techniques (14.0%), Radiological Techniques (6.2%), Anesthesia Techniques (13.4%), and Prosthetic Dental Techniques (10.8%).

Regarding the academic year, 35.6% were fourth-year students, 20.8% third-year, 14.6% second-year, and 29.0% first-year. With respect to screen time, 13.2% reported less than 2 hours/day, 22.2% used screens for 2–4 hours/day, 26.2% for 5–7 hours/day, and 38.4% for more than 7 hours/day. Females were significantly more likely than males to use corrective glasses or contact lenses (p < 0.001). The demographic characteristics and corresponding percentage frequencies are summarized in Table 1.

**Table 1:** Demographic characteristics of participants.

Age category	Male n (%)	Female n (%)	p-value
**19–20 years**	148 (35.3)	276 (64.7)	< 0.001
**21–22 years**	107 (33.9)	208 (66.1)	< 0.001
**23–24 years**	165 (39.8)	250 (60.2)	< 0.001
**College department**			
**Optics techniques**	256 (40.0)	385 (60.0)	< 0.001
**Medical laboratories techniques**	49 (30.4)	112 (69.6)	< 0.001
**Radiological techniques**	22 (30.6)	50 (69.4)	< 0.001
**Anesthesia techniques**	55 (35.5)	100 (64.5)	< 0.001
**Prosthetic dental techniques**	29 (23.2)	96 (76.8)	< 0.001
**Year of study**			
**First**	116 (34.8)	218 (65.2)	< 0.001
**Second**	60 (35.7)	108 (64.3)	< 0.001
**Third**	92 (38.2)	149 (61.8)	< 0.001
**Fourth**	152 (37.0)	259 (63.0)	< 0.001
**Screen time (mobile, tablet, laptop) (hours/day)**			
**< 2 hours**	100 (39.7)	152 (60.3)	< 0.001
**2–4 hours**	96 (37.5)	160 (62.5)	< 0.001
**5–7 hours**	105 (34.7)	198 (65.3)	< 0.001
**> 7 hours**	104 (30.3)	239 (69.7)	< 0.001
**Use of glasses or lenses**			
**Yes**	49 (16.6)	246 (83.4)	< 0.001
**Dry eye symptoms**			
**Blurred Vision**	280 (39.2)	435 (60.8)	< 0.001
**Burning**	188 (26.4)	525 (73.6)	< 0.001
**Pain**	182 (36.3)	319 (63.7)	< 0.001
**Eye Redness**	260 (37.0)	442 (63.0)	< 0.001

### Prevalence of dry eye symptoms

**Fig 1:**
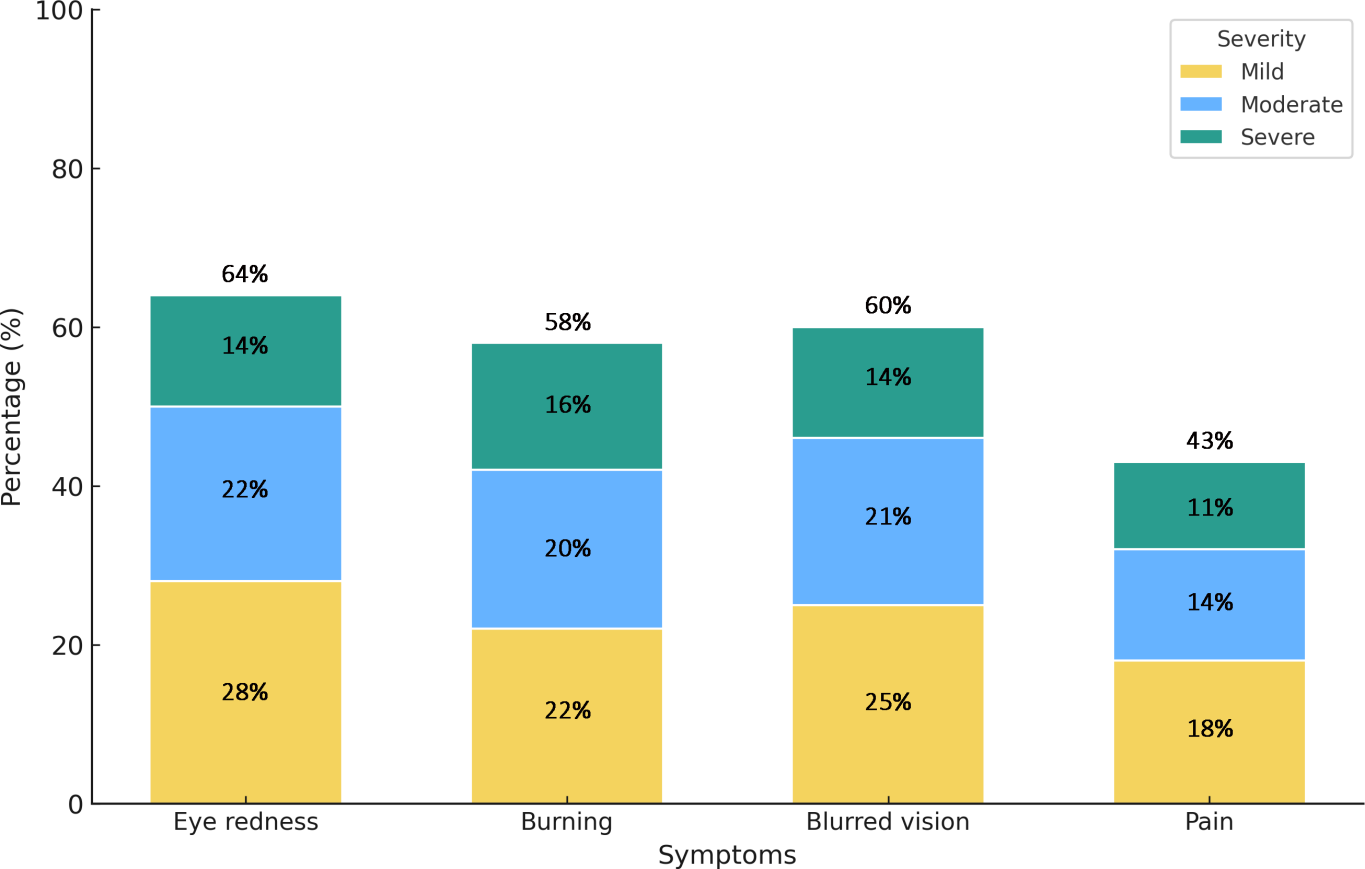
Severity distribution of dry eye symptoms among participants.

The analysis of dry eye symptoms among participants revealed that blurred vision, burning sensations, and eye redness were the most frequently reported complaints. In contrast, eye pain was the least common symptom. As illustrated in Fig 1, a considerable proportion of students experienced moderate to severe symptoms, particularly blurred vision, burning, and eye redness, confirming the significant burden of digital eye strain among participants.

Table 2 presents the distribution of DES symptoms across device types. Overall, symptom prevalence was relatively similar among devices, with slightly higher rates of eye redness and burning among laptop and smartphone users, respectively. Pain was less frequent overall. Statistical testing using the Chi-square test showed no significant differences between device categories (p > 0.05), though a trend toward higher redness and pain among certain groups was observed.

**Table 2:** Prevalence of DES symptoms by device type.

Symptom	Laptop (%)	Smartphone (%)	Tablet (%)	p-value
**Eye redness**	66.3	61.9	58.0	0.077
**Burning**	61.5	64.3	59.6	0.437
**Blurred vision**	63.3	61.9	62.3	0.925
**Pain**	45.3	54.3	51.1	0.054

### Symptom severity and screen time

**Fig 2:**
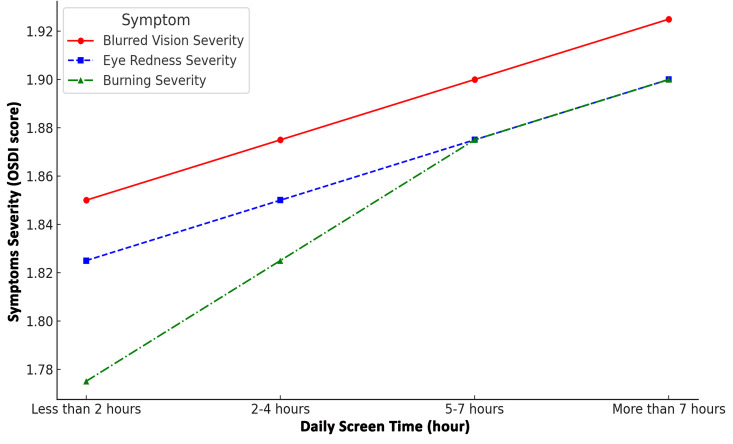
Association between daily screen time and severity of dry eye symptoms (mean OSDI score).

A clear dose-response relationship was observed between daily screen time and the severity of dry eye symptoms. As shown in Fig 2, increasing screen exposure was consistently associated with higher severity scores for blurred vision, eye redness, and burning sensations.

The correlation between screen time and symptom severity was analyzed using Spearman’s rank correlation coefficient (ρ), given the ordinal nature of both variables. The analysis revealed a moderate positive correlation between longer screen exposure and increased symptom severity (ρ = 0.47, p < 0.001).

Students who reported using digital devices for more than seven hours per day exhibited the highest symptom severity scores, confirming the significant impact of prolonged screen exposure on ocular surface health.

These findings highlight the critical role of digital device overuse as a major risk factor for ocular surface dysfunction and suggest the need for adopting preventive strategies, such as screen-time moderation and digital hygiene practices.

### Corrective measures adopted by students

Analysis of corrective measures adopted to manage dry eye symptoms revealed clear gender-based differences. As summarized in Table 3, female students were more likely to use lubricating eye drops and to take regular screen breaks, whereas male students showed a stronger tendency to adjust screen settings as a corrective approach. Notably, a considerable proportion of students in both groups reported taking no corrective actions to alleviate their symptoms, with statistical analysis confirming significant gender differences for most strategies and effect sizes ranging from small to large.

**Table 3:** Corrective strategies by gender with effect size.

Corrective Measure	Male (n, %)	Female (n, %)	p-value	Effect Size (Cohen’s d)
**Increasing rest periods**	80 (19.0%)	190 (25.9%)	<0.001	0.36 (Small)
**Eyedrops usage**	70 (16.7%)	223 (30.4%)	<0.001	0.72 (Medium)
**Changing screen settings**	150 (36.2%)	132 (18.0%)	<0.001	0.85 (Large)
**No corrective measures**	120 (28.1%)	189 (25.7%)	0.12	0.09 (Negligible)

### Factors for dry eye syndrome

**Fig 3:**
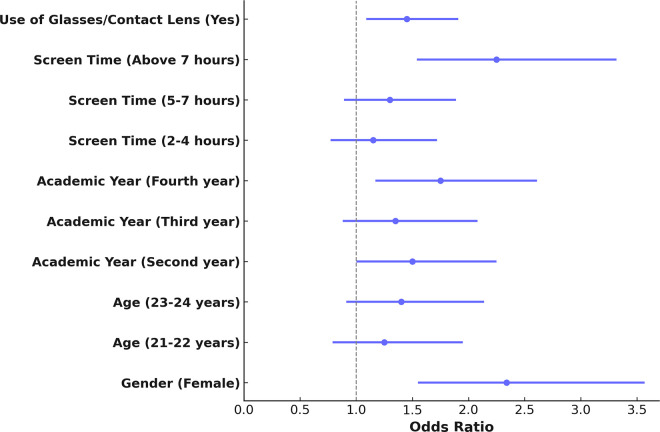
Statistical model binary logistic regression results for DES risk factors.

**Fig 4:**
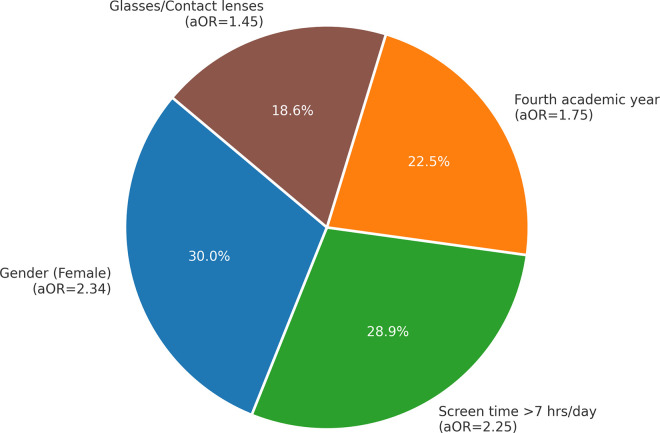
Key risk factors associated with DES (adjusted odds ratios).

The analysis of risk factors using binary logistic regression identified several significant predictors of dry eye symptoms (DES) among the participants. As shown in Table 4 and visualized in Figs 3 and 4, female gender emerged as the strongest predictor, with an odds ratio (OR) of 2.34 (95% CI: 1.56–3.56, p < 0.001), indicating that female students were more than twice as likely to develop DES compared to their male counterparts.

**Table 4:** Logistic regression analysis of DES risk factors.

Variable/ Category	Odds ratio (OR) (95% CI)	p-value
**Gender**		
**Males (Reference group)**	1.00	
**Females**	2.34 (1.56-3.56)	< 0.001
**Age**		
**19–20 years (Reference group)**	1.00	
**21–22 years**	1.25 (0.80-1.94)	0.32
**23–24 years**	1.40 (0.92-2.13)	0.12
**Academic Year**		
**First year (Reference group)**	1.00	
**Second year**	1.50 (1.01-2.24)	0.04
**Third year**	1.35 (0.89-2.07)	0.14
**Fourth year**	1.75 (1.18-2.60)	0.01
**Daily device usage (hours/day)**		
**Less than 2 hours (Reference)**	1.00	
**2-4 hours**	1.15 (0.78-1.71)	0.46
**5-7 hours**	1.30 (0.90-1.88)	0.15
**Above than 7 hours**	2.25 (1.55-3.31)	< 0.001
**Use of glasses or contact lenses**		
**No (Reference group)**	1.00	
**Yes**	1.45 (1.10-1.90)	0.01

Daily screen time exceeding seven hours was also a strong risk factor (OR = 2.25, 95% CI: 1.55–3.31, p < 0.001), confirming the significant role of prolonged digital exposure in increasing DES susceptibility. Students in their fourth academic year demonstrated a higher likelihood of experiencing DES (OR = 1.75, 95% CI: 1.18–2.60, p = 0.01), which may reflect cumulative screen exposure and increased academic workload. Additionally, the use of glasses or contact lenses was significantly associated with DES (OR = 1.45, 95% CI: 1.10–1.90, p = 0.01), suggesting that individuals with pre-existing visual challenges may be at an elevated risk of ocular surface dysfunction.

Fig 3 presents the logistic regression model results, highlighting the confidence intervals for each factor, while Fig 4 visually summarizes the contribution of the four key predictors to DES development. These findings align with global evidence indicating that female gender, extended daily screen time, advanced academic standing, and use of corrective lenses are among the most significant contributors to DES.

## Discussion

This study investigated the prevalence, severity, and associated risk factors of Dry Eye Syndrome (DES) among undergraduate students at Al Mustaqbal University (Iraq). The observed prevalence of symptomatic DES (62.0%) exceeds several international estimates for adult populations, underscoring the growing burden of ocular surface disease in younger cohorts [32,33]. The predominance of blurred vision, ocular redness, and burning sensations indicates meaningful effects on quality of life and academic functioning, consistent with prior reports describing daily life impairment among individuals with DES [34]. These findings align with global evidence that links the rising occurrence of DES in students to lifestyle shifts and sustained digital device use [35,36].

In the regional context, the present prevalence falls within the mid-range for the Middle East. In Saudi Arabia, a university cohort reported a 52.0% prevalence with blurred vision, burning, and gritty sensation as leading complaints [37], whereas the Jordanian ARB OSDI study documented a higher prevalence of 73.4% dominated by burning, dryness, and foreign body sensation [38]. The Syrian subgroup from the same ARB OSDI dataset showed 65.2% with redness, grittiness, and dryness as frequent symptoms [39], Turkish medical students exhibited a prevalence of 63.9% with light sensitivity, burning, and ocular pain most commonly reported [35]. Taken together, the Iraqi prevalence of 62.0% lies above that of Saudi Arabia yet below that of Jordan, consistent with regional variability in instruments, thresholds, and student digital workloads.

Beyond the Middle East, European cohorts continue to document substantial symptomatic burden. Polish university students exhibited a 57% OSDI-based prevalence, with symptom severity tracking prolonged digital display exposure [40]. Romanian medical student data further identify female sex, contact lens wear, and extended screen time as significant correlates of symptoms [41], while a Serbian student sample implicated prolonged device use and related ocular comorbidities [11]. Additionally, a 2025 nationwide survey in Spain reported a dry eye prevalence of 18.4% based on symptomatic criteria and 37.0% based on objective Schirmer testing, confirming a meaningful population-level burden even outside student populations [42]. These European findings underscore the consistency of DES prevalence and its strong behavioral correlates, particularly excessive daily screen exposure.

Outside Europe, Brazilian undergraduates exhibited high DES prevalence, largely associated with long daily screen hours and contact‑lens practices [43]. A 2025 South Korean national analysis estimated an overall prevalence of 8.9%, demonstrating female predominance and significant associations with prolonged screen use [44]. Such cross‑national observations situate the current results within a broader pattern whereby younger adults exhibit DES linked to contemporary educational and lifestyle pressures [35,40,41,43].

A clear dose–response pattern was evident between daily screen exposure and DES severity in this cohort; students reporting >7 h/day showed more than double the odds of symptomatic DES relative to peers with limited exposure, consistent with evidence from large population cohorts linking higher visual‑display terminal exposure to greater DES burden [45]. Mechanistic literature supports these associations: prolonged near work suppresses blink rate, increases incomplete blinks, and accelerates tear‑film evaporation, thereby destabilizing the ocular surface and amplifying symptoms [46,47]. Practical countermeasures, including structured micro‑breaks (e.g., the 20‑20‑20 rule) and adherence to basic digital‑hygiene guidance, have shown promise in improving comfort and tear metrics in interventional settings [48].

Gender‑related differences were prominent: female students were more than twice as likely to report DES than males, a pattern that echoes authoritative evidence implicating sex hormones and sex/gender influences in DES epidemiology and clinical expression [7,49]. The higher prevalence among females can be attributed in part to hormonal variations, particularly fluctuations in estrogen and androgen levels that influence tear film stability and meibomian gland function. Estrogen has been shown to promote ocular surface inflammation and reduce lipid secretion, while androgen deficiency impairs tear secretion and lipid layer integrity, leading to greater tear evaporation and instability. These mechanisms provide a biologically plausible explanation for the sex-specific pattern observed in this cohort [50–52].

The literature also identifies contact lens wear as a modifiable risk factor in student populations, often co‑existing with high screen exposure, creating opportunities for targeted counseling on lens hygiene, wear schedules, and fit optimization [41,53]. Corrective lens use was also associated with higher DES prevalence, possibly reflecting underlying refractive errors and visual strain common in Iraqi populations [54]. Taken together, these observations reinforce the importance of tailored education that addresses sex‑specific risks and behaviors [7,49,53].

Environmental conditions in Iraq may further compound the risk. In addition to urban air pollution and particulate matter [55], the arid and dusty climate characteristic of Iraq contributes to accelerated tear evaporation and ocular surface stress. Similar patterns have been documented in studies from countries sharing comparable climatic conditions, such as Saudi Arabia, Kuwait, Jordan, and Iran, where high temperatures, low humidity, and frequent dust exposure have been associated with greater rates of DES symptoms [56–60].

Reviews link airborne particulate matter and ambient pollution with ocular surface irritation and dry‑eye–related outcomes [55], while climatic analyses associate low ambient humidity and specific weather patterns with reduced tear stability and greater symptom severity [61]. Superimposed post‑pandemic learning models that increased reliance on e‑learning and prolonged screen exposure have likely expanded the symptom burden in university settings, consistent with meta‑analytic and cohort evidence [62,63]. Context‑specific prevention, such as indoor humidity management, exposure reduction during dust events, and structured device‑use breaks, is therefore justified for this population [55,61–63].

The widespread use of air conditioning (AC) systems in Iraq, particularly in educational and office environments, may further exacerbate the severity of dry eye symptoms. Air conditioning units lower indoor humidity and increase air circulation, which accelerates tear evaporation and destabilizes the tear film. This environmental factor has been recognized as a major contributor to ocular surface irritation and discomfort, especially in hot and arid climates where prolonged exposure to cooled, dry air is common. Similar findings were reported in regional studies from Saudi Arabia and the United Arab Emirates, which showed significantly higher OSDI scores among individuals frequently exposed to air-conditioned environments [57,64].

Overall, the data highlight the multifaceted nature of DES among university students and its growing public health importance. Given the high prevalence and the identified correlates (female sex, prolonged screen exposure, contact lens wear), institutional actions are warranted. Universities should implement digital‑hygiene programs, schedule periodic ocular screenings, and integrate preventive eye‑care content within curricula, in line with contemporary professional guidance and lifestyle‑focused recommendations for DES management [65,66].

## Conclusion

In this large, single-center cohort of Iraqi university students, symptomatic dry eye was highly prevalent (62.0% by WHS criteria) and scaled with daily screen exposure. After adjustment, female sex (aOR 2.34), screen time greater than 7 hours per day (aOR 2.25), fourth academic year (aOR 1.75), and use of glasses/contact lenses (aOR 1.45) were independent predictors of symptoms. Device-specific patterns were also observed, suggesting that ergonomics and viewing behavior may differentially affect the ocular surface. Taken together, these findings suggest a substantial and potentially modifiable burden of digital eye strain among young adults residing in an arid, dust-prone environment.

The findings of this study support campus-level prevention that prioritizes digital hygiene (e.g., scheduled breaks/20-20-20), ergonomic optimization, and periodic screening of higher-risk groups (heavy screen users, women, and contact-lens wearers). Given the cross-sectional design, reliance on self-reported instruments, and single-institution setting without objective tear-film testing, causal inferences and generalizability are limited. Future studies should incorporate longitudinal designs, objective ocular surface measures (e.g., tear osmolarity, TBUT/NIBUT, meibography), and environmental monitoring (humidity/particulate matter), as well as randomized evaluations of behavioral and ergonomic interventions. Such evidence will help define exposure thresholds and refine targeted strategies to reduce symptom burden and protect visual comfort and academic performance in student populations.

## Supporting information

S1 Data FULL DATA_Dry eye. The complete dataset used in the analysis of dry eye symptoms and associated risk factors.
(XLSX)
